# Immunogenicity risk assessment for tailored mitigation and monitoring of biotherapeutics during development: recommendations from the European Immunogenicity Platform

**DOI:** 10.3389/fimmu.2025.1581153

**Published:** 2025-05-22

**Authors:** Joanna Grudzinska-Goebel, Karin Benstein, Karien Bloem, Kyra J. Cowan, Boris Gorovits, Maria Jadhav, Melody Janssen, Vibha Jawa, Andrea Kiessling, Daniel Kramer, Arno Kromminga, Marcel van der Linden, Susana Liu, Gregor P. Lotz, Linlin Luo, Mantas Malisauskas, Céline Marban-Doran, Daniel T. Mytych, Elisa Oquendo Cifuentes, Susanne Pippig, Sandra Ribes, Myrthe Rouwette, Weiping Shao, Sophie Tourdot, Karin Nana Weldingh, Veerle Snoeck

**Affiliations:** ^1^ Preclinical Development, Pharmaceuticals R&D, Bayer AG, Berlin, Germany; ^2^ Translational Medicine Unit, Sanofi-Aventis Deutschland GmbH, Frankfurt am Main, Germany; ^3^ R&D Antibodies and Immunogenicity, Pharma and Biotech Services, Sanquin Diagnostic Services, Amsterdam, Netherlands; ^4^ Drug Metabolism and Pharmacokinetics, New Biological Entities, Research and Development, Merck KGaA, Darmstadt, Germany; ^5^ Bioanalytical Sciences, Regeneron Pharmaceuticals Inc., Tarrytown, NY, United States; ^6^ Pharmacokinetic Sciences - Drug Disposition, Biomedical Research, Novartis, Cambridge, MA, United States; ^7^ SciPot Consultancy B.V, Amsterdam, Netherlands; ^8^ Translational Medicine, Bristol Myers Squibb, Lawrenceville, NJ, United States; ^9^ Precliclinical Safety, Biomedical Research, Novartis, Basel, Switzerland; ^10^ Biolytics, BioNTech SE, Mainz, Germany; ^11^ Kromminga Consulting, Hamburg, Germany; ^12^ Bioanalytical Science, Genmab B.V., Utrecht, Netherlands; ^13^ Translational Clinical Sciences, Biologics & Immunogenicity, Clinical Bioanalytics, Pfizer, Vaughan, ON, Canada; ^14^ Roche Pharmaceutical Research and Early Development, Pharmaceutical Sciences, Roche Innovation Center Munich, Penzberg, Germany; ^15^ Regulated Bioanalytics, Merck & Co., Inc., Rahway, NJ, United States; ^16^ Non-Clinical Safety Research, H. Lundbeck A/S, Valby, Denmark; ^17^ Roche Pharmaceutical Research and Early Development, Pharmaceutical Sciences, Roche Innovation Center Basel, Basel, Switzerland; ^18^ Clinical Immunology Department, Clinical Development, Amgen, Inc., Thousand Oaks, CA, United States; ^19^ Drug Metabolism and Pharmacokinetics, Boehringer-Ingelheim Pharmaceuticals Inc, Ridgefield, CT, United States; ^20^ Scientific Affairs, Formycon, Martinsried, Germany; ^21^ Global Clinical Development, Hexal AG (a Sandoz company), Holzkirchen, Germany; ^22^ Bioanalysis & Protein Interaction Department, Byondis B.V., Nijmegen, Netherlands; ^23^ Integrated Bioanalysis, Clinical Pharmacology and Safety Sciences, AstraZeneca, Gaithersburg, MD, United States; ^24^ Pharmacokinetics, Dynamics and Metabolism, Pfizer Inc., Andover, MA, United States; ^25^ Non-Clinical and Clinical Assay Sciences, Novo Nordisk A/S, Måløv, Denmark; ^26^ Precision Medicine, Early Clinical Development & Translational Science, UCB, Braine-l’Alleud, Belgium

**Keywords:** immunogenicity, immunogenicity risk assessment, biotherapeutics, immunogenicity mitigation, bioanalysis, anti-drug antibody, immunogenicity de-risking, immunogenicity monitoring

## Abstract

Bringing safe and effective drugs to patients is of utmost importance for the pharmaceutical industry, with immunogenicity (IG) being a critical factor that influences both aspects. Biotherapeutics can elicit unwanted immune responses, potentially leading to (severe) safety implications, reduced patient benefits, and may result in termination of development. Therefore, understanding IG risks throughout drug development is essential for both drug developers and health agencies (HAs). The Immunogenicity Risk Assessment (IRA) facilitates the identification of IG risk factors and allows the establishment of effective mitigation and monitoring strategies. In this publication, the European Immunogenicity Platform (EIP) presents a comprehensive IRA framework aligned across pharmaceutical industry, emphasizing its significance in product development - from early de-risking to bioanalytical monitoring and mitigation measures during clinical trials. The EIP also provides an updated list of IG risks, offers distinct recommendations for assigning overall IG risk levels prior to the start of clinical development and highlights business considerations within this assessment.

## 1 Introduction

Biotherapeutics have the inherent potential to be immunogenic and to trigger immune responses directed against themselves (or endogenous counterparts), with consequences ranging from no effect to potentially diminishing treatment effectiveness and/or impacting patient safety. These may involve innate, cellular, and humoral immune reactions including acute or delayed hypersensitivity, infusion- related reactions (IRRs), injection site reactions (ISRs) and/or development of anti-drug antibodies (ADAs) (1, 2). This manuscript will focus mainly on questions related to humoral ADA immune response. ADAs can be formed mainly through a T-cell dependent mechanism or in rare cases through the T-cell independent pathway (3). In the T-cell dependent pathway, biotherapeutics are internalized by antigen-presenting cells (APC), degraded, and presented as peptides bound to the major histocompatibility complex (MHC) to T-cells, resulting in T- and subsequent B-cell activation which may trigger ADA formation. In the T-cell independent pathway, biotherapeutics with adjacent repetitive epitopes may directly crosslink B-cell receptors, leading to B-cell activation and ADA production. Importantly, the T-cell independent response with repetitive dosing predominantly leads to IgM responses. Due to the lack of T-cell help, which is required for the recombination process, the switch to high affinity IgG class maturation is rarely observed (3). The impact of ADAs can vary widely, ranging from antibody appearance without clinical significance to negative influence on pharmacokinetics (PK), pharmacodynamics (PD) and efficacy, ultimately resulting in loss of benefit, or safety events potentially leading to severe life-threatening conditions. Consequently, IG can halt or delay clinical development, which not only negatively affects patients but also poses significant business risks for drug development companies, including increased development costs, regulatory hurdles, reduced market size, resulting in competitive disadvantages. Thus, a risk-based approach is crucial to understand and anticipate potential IG consequences, to establish tailored mitigation strategies and address any immunological adverse event that may arise in association with the biotherapeutic treatment.

Rooted in the fundamental concept of risk management, the IRA has evolved over the past two decades now encompassing evaluations of various types of biotherapeutics and novel modalities. The IRA process can be subdivided into three different steps: i) identification of potential IG risk factors, ii) subsequent evaluation of the likelihood and potential IG consequences on safety, efficacy and business case, and iii) assignment of overall risk level (low/moderate/high). Consequently, the IRA guides the implementation of de-risking activities and defines the clinical IG testing strategy including additional monitoring for medium to high-risk molecules. It also justifies a leaner approach for low-risk molecules and informs the need for regulatory consultations during product development.

Despite extensive literature (**Table 1**), guidelines from HAs such as European Medicines Agency (EMA) (4, 5) and the U.S. Food and Drug Administration (FDA) (2, 6), and with significant industry experience, no harmonized and practical guidance exists on how to assess the overall IG risk for biotherapeutic drug candidates. The EIP has consolidated a framework for IRA based on the collected experience from different companies and from literature examples. Besides a comprehensive overview of relevant IG risk-factors, including novel aspects that evolved over the past years, the present publication provides an EIP-aligned approach on risk evaluation and the assignment of an overall risk level. Furthermore, guidance on the translation of the risk level to IG de-risking, mitigation and bioanalytical strategies throughout drug discovery and development of biotherapeutics, including biosimilars, is proposed.

**Table 1:** Literature overview on Immunogenicity Risk Assessment.

First Author, year	Type of publication	Immunogenicity Risk Assessment (IRA) recommendation	Bioanalytical (BA) monitoring strategy recommendation	Reference
Shankar et al., 2007	Perspective	A general strategy to broadly assign IG risk levels to biological drug products.	‘Fit-for-purpose’ BA scheme for low, mid, and high-risk products.	(7)
Koren et al., 2008	Industry Perspective	Recommendations for factors to be considered in assessing the risk of ADA related clinical sequelae.	Proposed BA testing strategy in clinical phases depending on low and high-risk category.	(91)
Jahn & Schneider, 2009	Review	In-depth regulatory discussion on key principles of systematic evaluation of IG during development of biologics.	Development of a suitable assay strategy to detect, quantify and characterize ADAs depending on clinical stage and IG risk.	(107)
Buettel et al., 2010	Review	Focus on the impact of IG on benefit/risk estimation of a therapeutic protein.	Not provided.	(108)
Buettel et al., 2011	Conference summary	Framework provided for risk estimation. Different approaches of combined risk identification, assessment/ADA testing, and mitigation.	Sampling strategy to provide information on ADA kinetic.	(109)
Chamberlain, 2011	Perspective	IRA process in connection with registration procedures.	Not provided.	(110)
Hock et al., 2015	Conference summary	Perspectives on adaption of IRA to reflect the complexity of ADCs.	Tiered approach and considerations to domain specificity and linker chemistry.	(48)
Kloks et al., 2015	Industry Perspective	Fit-for-purpose strategy for risk-based IG testing.	Testing strategies based on two categories, without and with expected potential to elicit ADA mediated severe clinical consequences.	(89)
Mytych et al., 2017	Commentary/perspective/advice	Event-driven IG testing strategy with no reference to IRA.	Low-risk therapeutic proteins supported with an event-driven IG testing strategy in early clinical phases, default ADA testing in pivotal studies.	(50)
Reinivuori et al., 2018	Review	Biosimilar recommendations for regulatory standards in EU and USA.	BA assay strategy including ADA incidence, titer and NAb assessment. Specificity testing of biosimilar vs. originator.	(111)
Chamberlain & Rup, 2020	Review	Early-stage IRA supports decisions concerning CMC strategy, prioritization of BA resource allocation and risk mitigation for clinical studies.	BA assay strategy including considerations for presence of endogenous counterparts.	(68)
Kernstock et al., 2020	Review	Theoretical case-study of IRA for a low-risk mAb. Additional information on potential actions in case of hypersensitivity reactions.	BA assay strategy throughout the different clinical phases. Considerations for use of biomarkers instead of NAb.	(112)
Mora et al., 2020	Case studies	IRA of pegylated proteins illustrated with two hypothetical case studies.	Corresponding BA strategy.	(46)
Sperinde et al., 2020	Review	IRA of receptor-Fc fusion protein and description of associated specific risk factors for this type of molecule.	Proposed detailed characterization of ADAs to a high-risk molecule and close monitoring of patients developing ADAs.	(10)
Bray-French et al., 2021	Perspective	Preclinical IG toolbox of *in vitro*/*in vivo* approaches for management of IG in early development.	Sampling and testing strategy for three risk categories.	(96)
Kroenke et al., 2021	Research paper	IRA to reflect the complexity of multi-specific biotherapeutics including a hypothetical case study.	BA assay strategy including choice of PK method, format, and an integrated PK/PD/ADA approach.	(31)
Jawa et al., 2022	Scientific paper	Risk assessment approaches at each stage of drug development using preclinical risk assessment outputs.	Recommendation of BA strategies based on risk and development stage.	(90)
Zhou et al., 2022	Review	IRA of bispecific Ab for cancer including review of the clinical relevance of ADA, advances in knowledge of tools and strategies for IG prediction, monitoring, and mitigation.	Not provided.	(11)
Harris & Cohen, 2024	Review	Overview of IG risk factors and recent advances in IG prediction strategies, with a focus on protein engineering used throughout development to reduce IG supplemented by proposals for clinical mitigation by co-medications.	Not provided.	(84)
Carter & Quarmby, 2024	Review	Overview of IRA and mitigation practices for protein therapeutics with a focus on identifying some of the major challenges with IG for which pragmatic approaches are provided.	Not provided.	(113)

## 2 Review of published data on immunogenicity risk assessment

Published literature provides extensive guidance on various aspects of IRA, including individual risk factors, examples for different biological modalities, and bioanalytical monitoring strategies for nonclinical and clinical studies (see **Table 1**). Our summary primarily focuses on the most relevant publications describing IRA and bioanalytical strategies aligned with HA guidelines (2, 4–6), while literature focusing on nonclinical/*in vitro* and *in silico* IG risk assessment assays (IVISIA) are not in scope of this overview table. Despite this comprehensive guidance, gaps remain in most of the current literature, particularly concerning the assignment of overall IG risk level and the integration of business risk considerations, clinical risk mitigation strategies, and effective communication of the IRA to HAs prior to the start of clinical development. Since the early recommendations were published (2, 4–7), there have been significant advancements in understanding and implementing IRA strategies, driven by evolving regulatory expectations, increased collaboration and data sharing within industry, and technological innovations. This review elaborates on these changes and highlights current best practices for IG risk management.

## 3 Immunogenicity risk identification

The initial step in the IRA process is the identification of all potential IG risks associated with the biotherapeutic. The IG profile is influenced by a multitude of factors categorized into product-, process-, patient- and treatment-related risks, demanding a careful multidisciplinary evaluation. During the initial assessment, typically performed before the candidate selection stage, not all aspects can be fully evaluated. At that stage, the assessment focuses mainly on product-related risk factors, such as the type of biotherapeutic modality, the mechanism of action (MoA) and target characteristics. Once potential candidates emerge, the assessment expands to sequence-related risks, including sequence origin and post-translational modifications. Patient-related risk factors, in terms of disease and/or immune status, and potential presence of pre-existing immunity, can also be considered if data are available. As the program advances from nonclinical stages to the start of clinical development, the assessment is supplemented by examining the process- and all remaining patient- and treatment-related risks.

Establishing a candidate IG profile in the absence of clinical data is challenging due to a complex interplay of risk factors such as product sequence, structure, MoA and drug formulation with patient’s disease status and treatment regimen. A direct translation of risk factor combinations into clinical outcomes is uncertain and often relies on existing knowledge from similar molecule classes or literature data. The EIP provides therefore a comprehensive and updated list of IG risks in **Table 2**. This is based on initial risk definitions and recent clinical findings with fully human or humanized biotherapeutic proteins exhibiting unexpected IG profiles, which were retrospectively connected to specific risks and their potential clinical implications, emphasizing the need for critical evaluation of these flagged liabilities as a foundational step of the IRA.

**Table 2:** Harmonized table on immunogenicity risk identification/assessment with literature references for higher risk.

Risk type	Risk Factor	Risk evaluation		Potential IG consequence(s)	Reference
		Low risk	Flag for higher risk		
Product	Sequence origin and degree of sequence foreignness	Fully human (germline) sequence with no polymorphism	Partially human (e.g. chimeric; homology in CDR to bacterial sequence; humanized; human sequence with polymorphism)	ADAs due to recognition of MHC-presented peptides as foreign	(5, 12, 16, 20, 81)
			Multi-specifics (higher number of foreign CDRs)		(11, 31)
			Neoepitopes in fusion molecules or conjugates		(10, 11)
			Cryptic epitopes		(11)
			Non-human (animal, bacterial, viral)	In addition to ADAs, risk for hypersensitivity reactions	(2, 5)
	MoA	Immunosuppressive No immune modulation	Immune stimulatory, synergistic or agonistic	ADAs in case of immunostimulatory/synergistic biotherapeutics. For agonistic MoA risk of ADA-mediated cross-linking of cell-surface receptors potentially leading to exaggerated pharmacology	(2, 5)
	MoA: Target	Soluble, monomeric	Soluble, multimeric	Immune complex formation between drug and soluble multimeric target leading to high incidence (>80%) of ADAs and loss of PK/PD, efficacy	(29, 30)
		Membrane protein no impact of receptor cross-linking on pharmacology	Membrane protein impact of receptor cross-linking on pharmacology	Altered/exaggerated pharmacology by ADA-mediated cross-linking of membrane receptors with safety consequences	(7, 32, 33)
			Membrane protein on APCs	Receptor-mediated uptake into APCs enabling MHC peptide presentation leading to ADAs	(21, 25, 41)
	MoA: Effector function (ADCC, ADCP, CDC)	No ADCC, ADCP, CDC Effector function leading to strong B-cell depletion	ADCC, ADCP, CDC without strong B-cell depletion	Exaggerated pharmacology, induction of necrosis creating an inflammatory environment and activation of adaptive immunity. However, afucosylated mAbs with enhanced ADCC in cancer show low IG but higher IRR incidences	(34–36, 38–40, 47)
	Targeted chemical modification	No targeted modification	PEGylation	PE anti-PEG Abs potentially leading to accelerated blood clearance, complement activation-related pseudo allergy (CARPA) or in very rare cases to anaphylaxis mediated by anti-PEG IgE Abs	(45, 46, 114)
			Fusion with linker and/or chemical structure e.g. toxophore or chelator etc.	Toxicity due to uptake of ADA-drug complexes into non-target tissue ADAs against hapten-like structures (vc-MMAE, DM1/4 or calicheamicin) reported only in very rare cases with no impact on PK, PD, efficacy, safety	(43, 47, 48, 115)
	Homology of biotherapeutic to endogenous counterpart	Endogenous counterpart with redundant function	Full or partial sequence homology to endogenous counterpart Endogenous counterpart with unique function	ADAs cross-reactivity to endogenous counterpart potentially resulting in autoimmunity phenotype	(2, 5)
Process	Aggregates	Low aggregate number (within process specific CQA limits)	Higher aggregate number (outside process specific CQA limits)	ADAs due to aggregate-mediated cross-linking of B-cell receptors, uptake into antigen presenting cells or triggering of immunostimulatory danger signals. However, a high threshold of aggregates needed (beyond that typically seen in marketed products)	(2, 49, 116)
	Level of impurities (e.g. host-cell proteins, lipids, DNA, bacterial contaminants)	Low (within process specific CQA limits)	Higher (outside process specific CQA limits) Presence of microbial impurities or impurities with homology to endogenous proteins	HCPs may be immunogenic, biologically or enzymatically active mediating •adjuvant activity of innate immune responses leading to ADA formation •hypersensitivity reactions when of microbial origin •proteolytic drug or excipient degradation resulting in ADAs •anti-HCP antibody formation potentially cross-reacting to homologous endogenous counterparts or remain without any consequences	(2, 49, 117–120)
	Leachables from containers or closures	Low (within process specific CQA limits)	Higher (outside process specific CQA limits) for tungsten, glass and metal particles, silicon oil	Leachable-induced ADAs due to protein denaturation, aggregation, modification	(2, 49)
	Degree of chemical modifications	Low (within process specific CQA limits)	Higher (outside process specific CQA limits)	Chemical modification (e.g. oxidation, deamidation, hydrolysis, isomerization) inducing drug aggregation resulting in ADAs	(2, 49, 121)
	Glycosylation patterns	Fully human	Partially or non-human (e.g. galactose-alpha-1,3-galactose, N-glycolylneuraminic acid (NGNA), high mannose content)	Non-human glycosylation triggering innate/adaptive immune responses Glycosylation pattern (e.g. high mannose content) enhancing uptake into APCs, leading to ADAs PE-ADAs leading to accelerated drug clearance, loss of efficacy or hypersensitivity (e.g. serum sickness) or anaphylaxis in case of anti-IgE abs	(122–125)
	Formulation	No adjuvant effect	Adjuvant effect not known or expected e.g. for polysorbate 20 or 80 Formulation impacting drug stability	Hypersensitivity including anaphylaxis. Formulation-mediated adjuvant activity of innate immune responses resulting in ADAs Formulation-mediated aggregation or degradation leading to ADAs	(2, 49, 52, 53, 126)
Patient	Basal patient immune status	Compromised or immunosuppressed	Activated or inflammatory immune system; autoimmune patients	ADAs in patients with activated, inflammatory, or autoimmune status of immune system	(2, 5, 41, 127)
	Genetic status	No classification		Identification of subpopulation at risk based on (retrospective) HLA analysis	(2, 5)
	Age	Elderly	Children/infants	Immunosenescence i.e. natural decline in immune system function with increasing age. However, exposure to a greater variety of antigens over the lifetime may increase the probability for IG	(5, 128, 129)
	Pre-existing anti-drug antibodies	Not detected	Detected	PE-ADAs described against PEG, non-human glycans, recombinant cytokines, growth factors, enzymes, pathogen similarity and new antibody fragment formats (e.g. Fabs, scFVs and VH domains) exposing cryptic or neoepitopes	(15, 32, 33, 56–58)
	History of Allergy	No allergy	Allergy(ies) present	Individuals with allergies have a hyperactive or dysregulated immune system with enhanced predisposition to develop IgE/Th2 immune responses against the drug or excipients	(2)
	Prior treatment with similar therapy w/o ADAs	Yes	No	ADA cross-reactivity to endogenous counterpart potentially resulting in autoimmunity phenotype	(5)
	Concentration of fully homologous endogenous counterpart	Really high abundance	None (null mutation) or relatively low abundance	For null mutation elevated probability of ADAs. For low abundance proteins weaker robustness of immune tolerance	(5)
Treatment	Dose level	High dose	Low dose	ADA impact on PK/PD more pronounced at lower doses	(2, 130)
	Treatment regimen: dosing frequency, schedule, length of treatment	Single dose	Multiple chronic dosing	Impact of ADAs on PK/PD, efficacy less likely for acute MoA upon fast clearing single dose compared to multiple chronic dosing	(2)
			Intermittent/episodic treatment	Longer drug holidays have been shown to correlate with increased ADA risk. Frequent dosing poses higher IG risk due to ‘prime and boost’ phenomenon often utilized in vaccine development	(130, 131)
	Route of administration	IV For mAbs only: SC	SC (for non-mAbs) < intramuscular or intradermal or inhaled	ADAs due to favorable uptake of biotherapeutic into APCs for all administration routes other than IV. However, for mAbs in many cases similar ADA incidence observed for SC and IV administration	(2, 60–63)
	Immunomodulating concomitant medication	Immunosuppressive comedication	Immunostimulatory comedication	Immunosuppressive co-medication may reduce whereas immunostimulatory co-medication may enhance ADA incidence	(132, 133)

### 3.1 The interplay between sequence, mechanism of action and target expression

Non-self-sequences present within biotherapeutics are one major determinant of IG (8). For monoclonal antibodies (mAbs) these sequences are mainly present in the complementarity determining regions (CDRs) (9). Modifications in other domains, for example in the CH2 domain to modulate or completely remove effector functions or introduction of linkers for generation of fusion proteins, may also introduce new T-cell epitopes (10, 11). A recent example of sequence-based risk of a drug with high titer ADAs in a portion of treated patients is the humanized mAb bococizumab targeting the soluble proprotein convertase subtilisin-kexin type 9 (PCSK9) (12). High titer ADAs were shown to have an impact on the long-term effective and durable decrease in cholesterol levels. There was a higher incidence of ISRs compared to other existing and evolving therapies, mostly in patients with ADAs. These results in light of the competitive landscape contributed to the discontinuation of bococizumab’s development (13). The murine sequence located in or near the PCSK9 binding CDR could be the cause of the high IG incidence (12). In addition, MHC-associated peptide proteomics (MAPPs) analysis showed a high number of potential CD4 T-cell epitopes (14).

In another example, pre-existing ADAs against brolucizumab, an anti-VEGF-A antibody single-chain fragment variable (scFv), were present in the majority of the patients, with increasing titers after treatment. A small portion of the ADA positive patients developed retinal vasculitis and/or retinal vascular occlusion as a severe adverse event (SAE). Although a direct mechanistic link between ADA detection in serum and the observed inflammation in eye tissue in patients is still lacking, it has been shown that the average IgG titers were significantly higher compared to the group without SAEs (15). The high IG incidence could, amongst other factors, be attributed to a linear sequence in the CDR H2 of the molecule shared with bacterial proteins (16). Both examples highlight non-human protein sequences within a human biotherapeutic as an important IG risk factor (amongst pre-existing immunity).

Furthermore, human mAbs such as golimumab and adalimumab have shown a moderate to high IG incidence resulting in reduced clinical efficacy, despite their categorization as low-risk molecules from a safety perspective (17–19). In these examples, ADAs are mainly formed against the CDR region (20). These drugs target tumor necrosis factor α (TNFα) and therefore in addition to sequence liabilities the MoA in terms of target characteristics may contribute to the high IG incidence. Besides its soluble form, TNFα also exists in a transmembrane form. It has been proposed that the high incidence of IG observed with anti-TNFα therapeutic mAbs could be partially explained by transmembrane TNFα-mediated drug uptake and antigen presentation on APCs (21). In contrast, the TNF receptor Fc-fusion protein etanercept showed very limited IG without clinical impact (22), which could be due to the absence of an immunogenic sequence (23) or potentially reduced binding of etanercept to transmembrane TNFα (24). A similar mechanism could explain the high IG incidence seen for mAb ATR-107, targeting the transmembrane protein IL-21 receptor present on APCs (25). Another contributing factor for the high IG of the anti-TNFα mAbs could be the size of the target-biotherapeutic complexes. Etanercept forms relatively small complexes with trimeric TNFα, while adalimumab and golimumab form larger complexes, where up to three drug molecules bind one trimeric TNFα (26–28). The influence of target properties on IG risk is further illustrated by bispecific mAbs targeting TNF and either Interleukin-17 or TNF-like ligand 1A which resulted in high ADA incidences (>80%) with clinical impact, that may have been driven by multi-specificity and multivalency of the biotherapeutic in combination with a multimeric soluble target leading to the formation of large immune complexes (29, 30). In addition, these multi-specific biotherapeutics may carry a risk for higher T-cell epitope content by the presence of multiple CDRs and/or linkers or regions not expressed in germline sequence of mAbs (11, 31).

For membrane receptors the risk of an altered and/or exaggerated pharmacology due to ADA-mediated receptor cross-linking needs to be considered. For example, the anti-TNFα receptor 1 (TNFR1) variable heavy chain (VH domain antibody) therapeutic, was cross-linked by the presence of pre-existing anti-VH autoantibodies and activated the TNFR1 receptor, thereby inducing symptoms of pro-inflammatory cytokine release, and inverting the pharmacology from an antagonistic into an agonistic function (32). In another example treatment with TAS266, a tetravalent variable heavy domain of heavy chain (VHH) activating the DR5 receptor to induce apoptosis in DR5 expressing tumor cells, was linked with hepatotoxicity during phase I clinical trials. Due to the high drug potency, it was proposed that binding of the drug-ADA complexes to the DR5 receptor expressed on hepatocytes resulted in enhanced drug activity, leading to apoptosis (33).

### 3.2 The contribution of ADCC and CDC activity

Immunomodulatory biotherapeutics have the risk of inducing immunotoxicity related to exaggerated pharmacology in both animals and humans. One of the major determinants of immunotoxicity is the design of the Fc portion of biotherapeutics to increase or minimize antibody-dependent cellular cytotoxicity (ADCC) or complement dependent cytotoxicity (CDC) in conjunction with the target specificity and target distribution within normal cells and tissues. ADCC and CDC activity may enhance the risk of an immune response to the biotherapeutic via stimulation of Fc gamma receptors (FcγRs) leading to direct cell killing, necrosis due to the cell-depleting MoA, the release of inflammatory mediators, and endocytosis of immune complexes via FcγRIIb and FcγRIIc. Internalization by activating FcγRs favors antigen (Ag) processing and presentation to T-cells, leading to activation of adaptive immunity (34–36). Ag internalization by FcγRIIb can also be recycled for presentation to B-cells, leading to T-cell independent ADA formation manifested as an IgM response (37). On the other hand, apoptotic cell removal and/or autophagy may promote an immunosuppressive environment and tolerance (34, 38).

Interestingly, afucosylated antibodies in oncology trials, which show higher ADCC potency, did not have increased ADA incidences but did exhibit higher IRR rates, consistent with the release of inflammatory mediators due to their MoA (39, 40). In contrast, the humanized mAb alemtuzumab targeting CD52 to deplete most lymphocytes via ADCC and CDC resulted in very high incidence rates of ADAs and neutralizing antibodies (NAbs) when treating patients with multiple sclerosis (MS). A complex interplay of different factors including the inherent biology of the molecule, the pattern of CD52 antigen tissue expression and the depletion/repopulation kinetics of immune cells may attribute to this incidence (41). Rapid lymphopoiesis or insufficient depletion of targeted lymphocytes while the drug is still present and/or reduced immune tolerance to the biotherapeutic are expected to contribute to the fast induction of ADAs. In contrast to patients with MS, ADA incidence in chronic lymphocytic leukemia (CLL) patients was very low. This suggests the contribution of additional risk factors such as the regimen (more intensified treatment schedule for cancer patients) or difference in patient disease status or genetics of people with MS, potentially predisposing them for generating immune responses in contrast to immunosuppression in tumor patients (41, 42).

### 3.3 The impact of targeted conjugation and modification

Another potential IG risk factor is associated with the attachment of non-protein components to the biotherapeutic, thereby forming new structural motifs. Antibody drug conjugates (ADCs) are designed to target a cytotoxic moiety to a cancer cell due to specific antibody binding to a target protein. Although theoretically ADAs can be formed to all different parts of the ADCs, thus far they are mainly directed against the antibody moiety (43). ADA development against ADCs increases the risk of adverse events, due to a higher potential for uptake of the ADA-ADC complexes by other (mainly immune) cells, and subsequent release of their cytotoxic drug payload in these cells (43).

Another example of targeted modification is the addition of polyethylene glycol (PEG) to biotherapeutics which was initially introduced to improve solubility, increase half-life and reduce IG. Presence of anti-PEG antibodies (IgM, IgG) may result in accelerated blood clearance leading to reduced efficacy or in hypersensitivity such as complement-associated pseudo-allergy when mast or granulocyte activation occurs, impacting safety (44). In addition, although rare, presence of anti-PEG IgE antibodies may induce anaphylaxis (44, 45). Recommendations for IRA of PEGylated proteins (46) and of ADCs (47, 48) have been published previously.

### 3.4 Manufacturing and product quality factors

Over the past years, progress in analytical methods and biologics manufacturing has significantly improved assessment of drug product quality and enabled setting more stringent control for critical quality attributes (CQAs) such as degree of chemical modification, aggregates and product and process derived impurities (including host cell protein content) (49). Consequently, an attributable impact of process-related impurities on the IG profile has been rarely observed lately (50) in particular for intravenous (IV) and subcutaneous (SC) drug formulations. However, challenges may remain for alternative formulations such as those required for example for inhaled delivery (51) or for long-acting injectables (52, 53). Therefore, concerns regarding potential impact of changes in CQAs on IG for IV and SC formulations has shifted to advanced stages in product development, such as when alterations in the manufacturing process occur like a manufacturing drug substance scale-up to commercial production, and/or change in manufacturing facilities. In cases where manufacturing changes result in CQAs exceeding the initially established quality control ranges with a defined nonclinical and clinical profile including IG, an overall risk assessment addressing aspects such as PK, PD/efficacy, safety and IG becomes necessary, during either pre-marketing or post-marketing stages. The IRA as part of the overall risk evaluation is essential to determine whether changes in the IG profile could result for the drug produced with the updated manufacturing process, and what additional consequences may occur along with potential clinical mitigation strategies to lessen impact to product safety and efficacy.

### 3.5 Contribution of pre-existing ADAs

The IG risk driven by pre-existing ADAs (PE-ADAs) cannot easily be differentiated from other potential contributing factors, such as presence of neoepitopes, linkers, MoA, and the antigenic target itself. Presence of memory B-cells for PE-ADA has been identified (15, 54), although clear evidence for boosting upon treatment with the biotherapeutic is lacking. PE-ADAs can form complexes with the drug, which can be taken up by the immune system (55). Consequently, this could lead to increased immune activation and enhanced clearance of the biotherapeutic, with a subsequent impact on efficacy and safety. There is extensive literature available on PE-ADAs for a variety of biotherapeutic modalities (56–58). Although the effect of PE-ADAs in an AAPS survey from 2013 on PD, PK, efficacy and safety was shown to be small (59), there are several examples where the PE-ADAs can impact the IG profile of the biotherapeutic in combination with other risk factors. Especially with the novel structural formats, like potential multi-specific products, the risk of PE-ADAs is higher due to the potential presence of neoantigens or exposure of cryptic epitopes (10, 11, 32). For example, with a bispecific molecule consisting of a scFv linked to an IgG molecule (IgG-scFv) the pre-existing IG directed to the scFv was considered to contribute to the high incidence of treatment emergent ADAs (56). As mentioned earlier, PE-ADAs against new antibody fragments may amongst other factors have contributed to the IG profile of brolucizumab (15, 16), the TNFR1 variable heavy chain (VH domain antibody) (32) and the tetravalent VHH TAS266 activating the DR5 receptor (33). PE-ADAs against PEG may also significantly impact the efficacy and safety profile of PEGylated biotherapeutics (44) or nanoformulations with PEG-moieties (52, 53) and should be considered within the IRA.

### 3.6 Considerations related to the route of administration

The administration route has been flagged as one of the treatment-related factors contributing to the development of IG. The IV route is associated with the lowest IG risk, whereas due to the potentially enhanced uptake mechanism of biotherapeutics into APCs other routes such as SC, inhaled, or intradermal may be associated with a higher IG risk (2). Though, for most full-length therapeutic mAbs, this has not been observed, as recent examples indicate similar IG rates between IV and SC administrations (60–63). In contrast, for other biotherapeutics, including antibody fragment-based modalities the SC route has been more immunogenic than IV. For example, two PSMAxCD3 immunomodulatory T-cell engagers, pasotuxizumab and JNJ-63898081, demonstrated IG incidence rates of 96.7% and 63%, respectively, upon SC dosing with clinical impact on both PK/PD and efficacy. Conversely, after IV administration ADA incidences were 0% and only 16.7% for pasotuxizumab and JNJ-6389808, respectively (64, 65). A follow-up analysis with pasotuxizumab has demonstrated that non-tolerant sequence-based epitopes potentially in combination with dose and dosing frequency contributed to the robust and clinically impactful ADA response after SC administration while excluding drug product CQAs and patient immune status (66). Limited data are available for biotherapeutics administered via the inhaled route, and there are individual examples where high and low IG have been observed (67). Overall, the evaluation of IG risk associated with the route of administration should be performed in context with other risk factors including product amino acid sequence, MoA, dose and dosing regimen, as well as product quality-related risk factors such as aggregates and formulation.

### 3.7 Biotherapeutics with endogenous counterpart

The IG risk associated with biotherapeutics, including peptides with amino acid sequence homology to an endogenous counterpart, is multifaceted as the sequence homology, posttranslational modifications (i.e. glycosylation) and the expression level of the endogenous protein must be evaluated in addition to all other factors (2, 5, 10, 68). For fully homologous biotherapeutics the likelihood of IG may be lower due to immune tolerance to the endogenous protein, although immune tolerance can be broken by treatment with the biotherapeutic. Indeed, individuals are not equally tolerant to all endogenous proteins, and the robustness of the immune tolerance to a specific endogenous protein depends on many factors, with the abundance of the endogenous counterpart being particularly important. When the primary amino acid sequence of the biotherapeutic deviates from the endogenous counterpart (69), or if patients completely lack protein expression (null mutation), the probability to develop an immune response impacting efficacy and potential safety is elevated. Safety consequences should also be considered when residual amounts of the endogenous counterpart are expressed. These arise when ADA-mediated functional neutralization of the biotherapeutic cross-reacts with the endogenous counterpart, resulting in an autoimmune disease potentially worsening patient disease status, especially in cases of a unique functionality. Consequently, HAs recommend the assessment of the impact of IG for biotherapeutics with an endogenous counterpart, while considering the extent of sequence identity (including the extent of polymorphisms in the relevant patient population), redundant or nonredundant physiological function of the endogenous protein, its expression level in patients, and the robustness of the anticipated immune tolerance (2).

Significant impact of IG has been observed in the context of recombinant coagulation factors FVIIa (70, 71) and FVIII (72), recombinant erythropoietin (73), modified thrombopoietin (74) as well as human acid a-glucosidase (75). In contrast, for insulin or GLP-1, clinical data has shown that the ADA response has limited impact on treatment effect and without safety concerns (76, 77). This retrospective evaluation was incorporated into the FDA draft guideline for biosimilar insulin products. This guideline allows for a waiver of comparative clinical IG studies if a comprehensive data package with a robust analytical assessment and clinical pharmacology study demonstrates comparability, and an IRA justifies little or no residual uncertainty regarding the clinical impact of IG (78).

## 4 Assignment of overall immunogenicity risk level prior to start of clinical development

**Figure 1:**
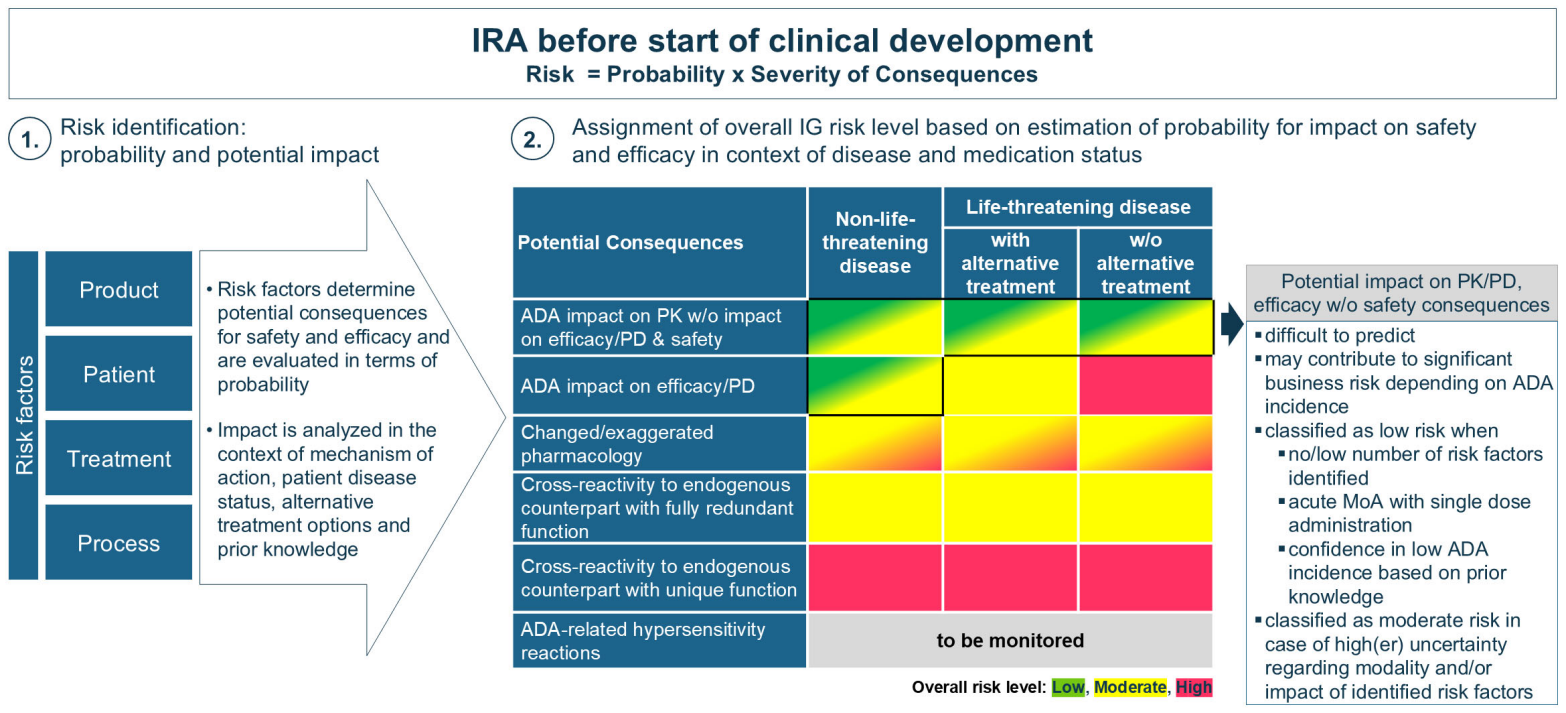
Assignment of overall IG risk level (low, moderate or high) prior to start of clinical development. After risk identification the potential impact of IG is evaluated in relation to the disease status and available alternative medication to assess its potential severity. Since the presence of alternative medications only affects the IG risk category for life-threatening diseases, this differentiation has been excluded for non-life-threatening diseases. This evaluation is influenced by existing knowledge. A high-risk category is assigned when severe safety consequences are anticipated. A low-risk categorization is recommended in case of sufficient certainty that no or little impact of IG will be observed otherwise a moderate category is applicable (as further explained in the box).

Following risk identification, the totality of IG risk factors are evaluated for their probability of occurrence and potential implications on efficacy and safety. In the absence of clinical data, this analysis is supplemented by existing knowledge from the same or similar molecular classes and literature data. The EIP presents a consolidated proposal for translation of the anticipated clinical consequences, in relation to the disease status (life-threatening disease or not) and availability of alternative medication, into an overall IG risk level (low, moderate or high) assigned prior to start of clinical development, as depicted in **Figure 1**. Alternative medication refers to standard of care options, as well as to other drugs that are either in development or already on the market. For further clarification, in oncology (life-threatening disease), patients often transition quickly to the next clinical trial if no early benefit is observed from the therapy, indicating that alternative treatment options are available. It is important to note that the assignment of a different risk category may be justified when additional knowledge or data from clinical studies becomes available, or if sufficient certainty exists. Further, it is essential to clarify that the IRA is distinct from the overall benefit-risk evaluation of a biotherapeutic program. The IRA focuses specifically on the potential impact of IG on safety and efficacy, enabling the definition of appropriate IG mitigation and monitoring strategies. In contrast, the benefit-risk evaluation encompasses a broader analysis that weighs the overall therapeutic benefits against all associated risks, including but not limited to IG. The balance between benefit and risk is also affected by the disease context (life-threatening or not) and the availability of alternative treatments. For example, in non-life-threatening conditions there may be less tolerance for certain risks, including IG. Conversely, in life-threatening conditions there may be a higher acceptance of risks to save patients’ lives, and risk tolerance may further increase when no alternative therapies are available. Consequently, programs with a high IG risk and confirmed anti-drug antibody (ADA) incidence may still be marketed despite impacting efficacy and/or safety, while lower-risk programs with similar IG might be halted or delayed, especially when more effective and safer alternatives are available.

To assign an overall IG risk category, the evaluation focuses on the identification of potential safety implications, irrespective of their probability. The highest concern applies to the substitution of endogenous proteins, particularly for those with unique, non-redundant functions. For endogenous proteins with a unique function, a high-risk category is recommended unless justified otherwise (e.g. GLP-1 and insulin). In cases where redundancy for the protein exists endogenously and there is sufficient knowledge and certainty of functional compensation, the overall IG risk level is moderate. It is important to underline that consequences of neutralization of an endogenous protein with partial redundant function may not always lead to immediate clinical symptoms (2), emphasizing that a high-risk categorization should be considered in case of uncertainty towards full functional redundancy.

Another concern emerges from ADA-mediated altered or exaggerated pharmacology impacting safety, particularly for biotherapeutics targeting membrane receptors (32, 33) or theoretically, for modalities with a short-half-life (e.g. peptides). The severity of these safety implications strongly depends on the MoA, which is further assessed in relation to the disease (whether life-threatening or not) and the availability of alternative therapies enabling the categorization into moderate or high risk.

IG-mediated loss of efficacy in life-threatening conditions (e.g. enzyme deficiency diseases) poses a significant safety risk and is assessed as moderate or high, depending on the availability of alternative treatment options. In contrast, IG impact on efficacy in non-life-threatening diseases is generally without safety implications but may imply a significant business risk depending on the extent observed in the target population. However, predicting the potential impact of IG on PK and/or efficacy from the type or number of identified risk factors is extremely difficult and remains uncertain. If risk identification does not highlight any significant concerns, or when ADA impact on efficacy can be excluded based on the drug’s MoA (e.g. if ADA-impact on PK is delayed compared to an immediate PD effect with single dose administration), or upon available prior knowledge about the molecule or similar compounds, then the biotherapeutic program may be classified as low IG risk. Conversely, if multiple risk factors are identified particularly when related to primary sequence, MoA, or a patient-related risks and a considerable degree of uncertainty remains about the potential effects on PK and/or PD/efficacy, a moderate IG risk category may be more appropriate. This allows to account for the uncertainty, to frontload analysis and gain insights into the IG profile of a biotherapeutic early in development. It facilitates strategic decision-making and implementation of clinical mitigation to increase the chances of success in clinical development, particularly since available literature illustrates how IG negatively affecting PK and/or efficacy in the absence of safety concerns may lead to halting clinical development. Assigning a moderate risk category may also be warranted when first-in-human studies are conducted in patients and when accelerated development is anticipated (for instance, moving directly from Phase I/II into pivotal studies) to obtain early insights into the IG profile (and IG assay performance). Furthermore, early IG assessment may be crucial for achieving a best-in-class market position, especially when developing less immunogenic alternatives than competitors. In contrast, when companies use a different risk model where safety is the main basis of the IG risk evaluation a low-risk category is assigned to programs where no impact on safety is anticipated. In these instances, the IG profile is evaluated in later studies (Phase II or pivotal) which are performed in the targeted patient population. It should be highlighted that not analyzing samples in Phase I is only acceptable if no unexpected findings related to PK, PD/efficacy (if applicable), or safety events are detected. Consultation with HAs on this strategy prior to start of clinical development is highly recommended.

## 5 Hypersensitivity reactions

Besides the ADA-mediated impact on PK, PD, efficacy and safety, hypersensitivity reactions may also impact patient’s safety, although they may not always be mechanistically linked to ADA formation (2). These immune-related adverse events (irAE) include acute or delayed hypersensitivity reactions (including anaphylaxis) and cytokine release syndrome (CRS) (1, 2). The safety consequences of irAE may vary widely and are often unpredictable in patients administered biotherapeutic products. Therefore, a high level of caution should be maintained for clinical events that may originate from such responses, with appropriate management strategies available in case they occur, even if the initial risk assessment suggests a lower risk of IG. Depending on the severity and the observed frequency of these safety events, characterization and elucidation of the underlying pathophysiology for irAE is encouraged, because this information may identify patients at risk and provide insight into potential mitigation strategies (2, 79). However, it may not always be possible to identify a specific immunologic mechanism as the basis of an adverse event. Moreover, the presence of ADAs is not necessarily predictive of anaphylaxis, other hypersensitivity reactions or CRS which is often driven by the MoA of the molecule (2, 80).

## 6 Immunogenicity risk assessment during clinical development

Once clinical data become available, the initial IG risk category may be adjusted to reflect either an increased or decreased risk level, enabling the adaptation of the monitoring and mitigation strategy, as needed. The overall IG risk level in the context of disease status (non-life-threatening or life-threatening) may be different in healthy volunteers and diseased subjects. First-in-human studies are often conducted in healthy volunteers which may or may not represent the target population. If later clinical data in the target patient population reveal a low incidence of ADAs and/or no significant impact on PK, PD, efficacy, or safety, the program may be re-classified as low risk, regardless of the patient’s disease state or the available alternative medications. However, if severe safety implications arise and decisions regarding patient treatment or discontinuation are based on the presence of ADAs, a high-risk category is recommended.

## 7 Immunogenicity risk assessment impact on nonclinical mitigation and monitoring

**Figure 2:**
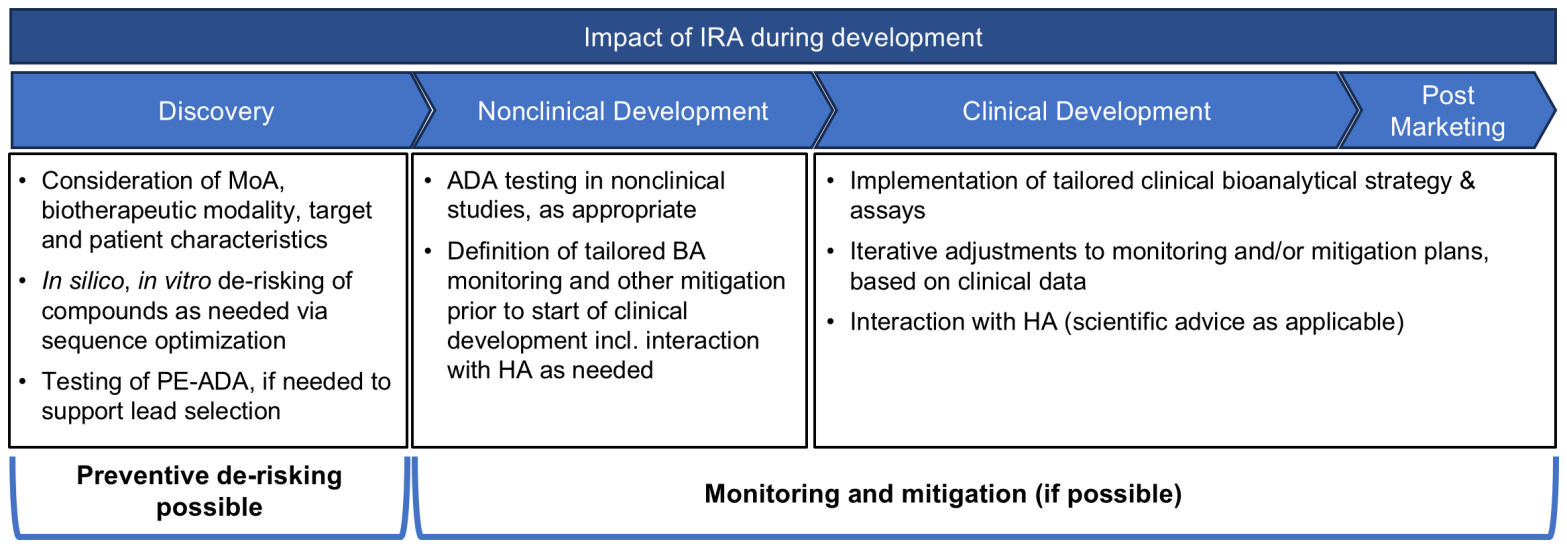
Impact of IRA during product development. During concept phase, it is recommended to start the IRA and incorporate the IG risks into the earliest discussions on molecule design. *In silico* prediction tools are often employed across the industry, to ensure that at minimum there is a de-risking of the amino acid sequence of the potential leads. For high-risk molecules, or new platforms, PE-ADA tests can be implemented. Additional *in vitro* tests, such as MAPPs, T-cell activation and B-cell epitope prediction assays and tools, can be utilized to confirm observations from the *in silico* tests, and to gate any de-immunization activities as needed. After candidate selection, the IRA guides the monitoring and mitigation strategy throughout development.

The outcome of the IRA defines the preventive and reactive mitigation of IG risks and the bioanalytical IG monitoring strategy throughout development (**Figure 2**). Although not all IG risk factors can be assessed during the initial product development stages, early IG risk identification offers significant advantages for candidate selection. The IRA may guide the choice of the most appropriate biotherapeutic modality, the evaluation of target properties, the analysis of PE-ADA, and the optimization of protein sequences to reduce potentially immunogenic T-cell epitopes. This approach aims to minimize the IG potential while also considering other important non-IG-related characteristics prior to final lead candidate selection. Companies may follow different de-risking strategies. Some prioritize de-risking based on the IG risk level conducting no or minimal activities for low-risk molecules. Others prefer to mitigate IG risks for all molecules regardless of the IG risk classification. The latter strategy helps to address the potential negative impact of IG on PK and/or efficacy, which could lead to clinical development discontinuation, as reported in literature. For moderate- or high-risk molecules, the IRA allows adaptation of mitigations strategies tailored to the specific IG risks identified. Understanding the differences in sequences and functional characteristics, potentially impacting the IG profile, between various therapeutic modalities is important. These may include target binding properties, presence of multimeric binding sites, ability to bind complement or Fc-gamma receptors, method of half-life prolongation, domain structure, glycosylation pattern, and the risk of altered pharmacology by target/molecule structure and function. For candidate ranking and selection several *in silico* and *in vitro* methods can be used as comprehensively depicted by Ducret et al. (81) and others (82–85). Furthermore, when feasible sequence optimization may allow a rational reduction in immunogenic T-cell epitopes, pre-existing antibody binding, immune complex formation, product internalization and pro-immunogenic aggregate formation without compromising important pharmacological properties. For high-risk molecules, or new platforms, PE-ADA testing can be implemented to assess any general population risks, even before the exact patient populations have been defined. Furthermore, PE-ADA tests can be conducted on the lead candidates as needed in later stages or for identified patient population matrices. However, sometimes certain IG risks must be accepted to support an essential pharmacological aspect of the molecule. Note that some companies include a summary of their nonclinical de-risking efforts (such as the *in silico/in vitro* T-cell assessment) in the IRA as part of the IND submission.

In nonclinical studies, the IG assessment is conducted to support data interpretation by explaining unexpected loss of exposure, efficacy, or immune-related safety findings (86). As outlined by Lauren et al. (87), the IRA facilitates estimating whether any IG-mediated impact may be observed, and together with additional business risk considerations helps to decide whether ADA method development needs to be prioritized. A lean ADA method validation is proposed for this purpose. It is well acknowledged that ADA frequency and effects in non-clinical *in vivo* studies cannot predict human IG due to species differences such as variations in the major histocompatibility complex (MHC) and T-cell receptor (TCR) repertoires. However, it should be considered that in exceptional cases, the causality of IG-related consequences on safety or even PK/PD, efficacy might be extrapolated from animals to human, when the MoA of the biotherapeutic target expression and function, and the binding properties between target and biotherapeutic as well as other aspects of target biology, are similar between species (88). In these cases, it might be worthwhile to identify the causality of high and/or impactful IG in animals prior to progression into human and to implement mitigation and monitoring strategies by customizing the bioanalytical strategy in clinical studies (e.g., more frequent IG measurements, early assessment of neutralizing ADAs, implementation of markers of ADA-related activation of innate immune pathways or adaptive immune stimulation leading to increased inflammation, unwanted ADA-mediated cell destruction or vascular damage).

## 8 Tailored risk-based clinical immunogenicity monitoring and sampling strategy

At the start of clinical development, the IRA compiles the IG risk profile and summarizes preclinical de-risking efforts. This assessment links the identified risks to an IG bioanalytical monitoring strategy, detailing assay types, sample collection, analysis schedules and supporting clinical endpoint choices for PK, PD, efficacy, and safety. The EIP has previously recommended to consider two testing categories: one for biotherapeutics with lower potential risk of ADA mediated events and one with the risk of severe consequences (89). These categories allow a fit-for purpose testing strategy focusing on a) monitoring design based on anticipated ADA mediated clinical consequences, and b) delivering ADA data to inform proper decision-making during drug development (89). Although the two testing categories remain important, the increasing complexity of biotherapeutics and the evolved understanding of IG risk necessitate a more granular link between the three IG risk categories (low, moderate, high) and testing strategies.

The definition of the bioanalytical strategy should directly correlate with the IRA, as summarized in **Table 3**. Nonetheless, many companies still follow a three-tiered analytical approach and frequent sampling strategies in all patients and studies without considering the possibility to define a tailored BA strategy based on the IRA (50). Instead, for biotherapeutics with low IG risk in Phase I studies (often conducted in healthy volunteers), an event-driven IG testing strategy should be considered (50). This approach involves banking samples and analyzing them only when altered PK, PD/efficacy (if applicable), or any safety-related events occur (89, 90). Consultation with HAs is advisable prior to implementing this strategy, for instance, by clarifying this approach as part of the IRA that includes the testing and monitoring strategy presented at the start of clinical development. The proposed approach is similar to prior recommended practices, which involved batchwise ADA analysis at study end, which can be implemented alternatively.

**Table 3:** Immunogenicity monitoring and sampling strategy.

	Phase I			Phase II			Phase III/Pivotal study		
Monitoring and Sampling Strategy	Low risk	Moderate risk	High risk	Low risk	Mode-rate risk	High risk	Low risk	Mode-rate risk	High risk
**ADA testing strategy**	Assay available	Analyze samples	Analyze samples (fully validated assay)	Analyze samples		Analyze samples (fully validated assay)	Analyze samples (fully validated assay)		
**ADA characterization:** **Neutralization** **Other: domain specificity, cross-reactivity to endogenous counterpart**	Not needed	Assess the need and consider reagent generation for NAb and additional characterization assay development	NAb for high risk is often expected. Consider integrated (active) PK/PD/ADA as potential alternative Assess need and selection of relevant assay if of added value	Not needed	NAb evaluation to be considered for moderate risk, expected for high risk Consider integrated (active) PK/PD/ADA strategy instead of NAb Assess need and selection of relevant assay based on Phase I data if of added value		Consider integrated (active) PK/PD/ADA strategy instead of NAb	NAb evaluation expected for moderate and high risk Consider integrated (active) PK/PD/ADA strategy instead of NAb	
							Assess need and selection of relevant assay based on Phase I/II data if of added value		
**Timing of ADA analytics**	Collect and hold, analyze in case of unexpected PK, PD/efficacy (if applicable) or safety–related AE	End of study	At least at the end of each dose level Close monitoring prior to next drug administration	End of study		Batch wise throughout study Close monitoring	End of study (recommend to frontload analysis ahead of study end)		Batch wise throughout study Close monitoring
**ADA sample collection frequency**	Baseline, end of cycle/dose tier (based on dosing)	Baseline, end of cycle/dose tier, selected timepoints at 7–14 days and 3–6 weeks based on regulatory requirements and project needs and EOS	Baseline, onset of ADA response such as Day 7–14 after first exposure, end of cycle/dose tier, for consecutive cycle/dose tier pre-dose and EOS Frequent sampling through all stages. Post-study follow-up sampling required for high-risk with serious safety consequences from ADA Consider use of other samples, i.e. PK/PD sample for additional ADA analysis In disease population collect higher volume of pre-dose samples (at least 2ml)	Baseline, selected timepoints at 7–14 days and 3–6 weeks based on regulatory requirements and project needs and EOS		Frequent sampling through all stages	Less frequent sampling than Phase I & II		Frequent sampling through all stages and EOS Post-study follow-up sampling required for high-risk with serious safety consequences from ADA
	*Ad hoc* samples in case of SAE as part of safety assessment								
**Overall strategy**	Engage with HAs to align on IG sampling and testing								

EOS, end of study (approximately 5 half-lives after last drug exposure (6)).

For moderate risk biotherapeutics, the ADA testing should be conducted throughout clinical development, but additional ADA characterization assays such as NAb or ADA domain specificity assays (for complex chimeric molecules or those engineered with specific targeted modification), may not be necessary for non-pivotal studies. The generation of reagents can be frontloaded during Phase I to expedite development of ADA characterization assays if needed. If an IG risk is identified that requires more testing, a fit-for-purpose approach can still be followed to extend the testing strategy. For example, if ADAs against one domain are linked to a potential safety and/or efficacy risk, domain specificity assessment can be considered.

In cases where the IRA, based on evaluation of all IG risk factors, results in a high-risk category, a tailored IG monitoring strategy with additional ADA characterization assays (focused on the risk-based demand) and more frequent sampling is mandatory even at early phases (7, 91–93). This may consist of a PK/PD testing strategy that includes appropriate safety biomarkers, along with a more frequent and *ad hoc* ADA and NAb sampling, and/or sampling for further characterization (domain specificity or cross-reactivity to endogenous counterpart) if of added value. A NAb assay may be valuable if the appearance of NAbs correlates with safety consequences, but it may not be helpful when the high-risk category was assigned due to ADA-mediated exaggerated pharmacology. Proper wording in the clinical protocol should allow the use of residual PK samples for NAb analysis or other additional ADA characterization analyses. Pre-treatment baseline samples should have sufficient volume to establish such additional ADA characterization assays. Implementing an IG testing strategy with expanded ADA characterization assays from the beginning of clinical development for high-risk molecules provides data that may correlate with ADA-mediated safety signals and inform appropriate and timely mitigation and intervention strategies (94).

Overall, a flexible investment of time, assays, and costs in IG testing strategies, based on risk categories (low, moderate, high), allows the project team to prioritize essential activities, and ensure appropriate resource allocation (95). For example, the level of investment for ADA assay development and sample analysis might be staggered based on the IG risk (96) and the clinical development phase. A fully validated ADA method is typically implemented in pivotal clinical studies (6), except for high-risk molecules where full validation may be considered in earlier phases. This ensures that assays accurately detect ADA with the required sensitivity and specificity, allowing for appropriate identification of patients at risk. Furthermore, it is increasingly considered common for companies to determine the ADA response magnitude using the signal to noise (S/N) ratio rather than titer determination (95, 97, 98). Continuous S/N assessment is independent of risk class, and its suitability for implementation should be data-driven and depends on assay characteristics and dynamic range. Upfront consultation with HAs prior to pivotal studies on full implementation of S/N approach instead of titer is recommended.

A critical ADA characterization tier is the evaluation of the neutralizing effect, which is expected by HAs as monitoring for pivotal studies. However, in many cases, a standalone NAb assay cannot evaluate the neutralizing potential of ADAs with sufficient sensitivity during drug treatment. This challenge is particularly pronounced with cell-based neutralizing assays. Furthermore, the results of these assays are not necessarily correlating with the clinical relevance of the NAbs as the drug concentrations used in the assay may not represent physiologically relevant drug concentrations. In recent years, more sophisticated assay platforms have enabled the development of improved PK and PD assays. The use of such assays may provide a better evaluation of the neutralizing effect of ADAs than a NAb assay. Therefore, it should be examined whether the integrated evaluation of drug concentration (PK) and effect parameters (PD) and presence of ADAs may be more clinically relevant for assessing the neutralizing effect of ADAs than the classical *in vitro* NAb assay. In absence of PD markers, an active PK assay is recommended. This may be particularly relevant for low-risk molecules that have no safety consequences and exhibit low impact ADAs. The scientific rationale for using this approach in pivotal studies and for high-risk projects should be discussed with HAs.

Irrespective of IG associated risk, sample collection should occur at baseline and at the end of each dose tier/cycle, with *ad hoc* samples collected in case of serious adverse events (SAE) as part of the safety assessment at every stage of clinical development. A scheduling of additional sampling with dosing to monitor early IgM responses (7–14 days after first exposure), IgG responses (3–6 weeks after first dose) and an end-of-study (EOS) sample (approximately 5 half-lives after last drug exposure) is recommended at early stages, according to FDA guideline (6). However, for low and moderate risk molecules less frequent sampling may be appropriate. Blood collected from placebo group can be used as disease baseline, but it is not recommended to analyze for ADAs in the non-treated placebo study participants (95). When PE-ADAs have been detected in high prevalence, analysis of placebo samples may be considered to assess its natural fluctuation. For low-risk projects with a large Phase III program and/or a large pivotal trial, considerations could be made to only collect and analyze for ADA in a representative subset of the study participants. The subset of e.g. 1000 dosed study participants should be selected to represent the ethnicity and disease spectrum in the clinical studies. In such instances, residual PK samples collected in all study subjects can be planned as back-up, in case of an unforeseen need for ADA analysis. The approach should be aligned with HA prior to study start. The EIP has previously provided recommendations on when to extend ADA monitoring beyond study end, for patients that developed an ADA response against high-risk biotherapeutics (99), emphasizing that decisions on follow-up sampling should be based on safety consequences and individual patient’s benefit. Ultimately, while ADA evaluation during treatment-free periods is important, continued monitoring may not be necessary if safety concerns are minor or resolved.

The EIP highlights that a tailored approach for biotherapeutics must prioritize patient safety at all times and recommends aligning the IG testing strategy with HAs before implementation. By integrating an IRA-guided IG testing strategy, pharmaceutical industry can effectively address all IG risks with optimal resource allocation to develop safe and efficacious biotherapeutics.

## 9 Immunogenicity risk assessment impact on clinical mitigation beyond bioanalytical monitoring

When the IRA indicates an IG risk anticipated in humans which could not be de-risked, appropriate clinical mitigation must be implemented, which may require monitoring beyond the classical bioanalytical analysis of ADAs and NAbs. For example, patients at risk of developing severe safety consequences to IG may be excluded from studies, or the dosing frequency adjusted to avoid intermittent dosing, if feasible from a pharmacological perspective. For mild to moderate IRRs, slowing the infusion rate or using a lower priming dose followed by higher subsequent dosing may be considered (80), whereas in cases of severe IRRs, the infusion must be stopped. Re-introduction of treatment must be carefully evaluated in terms of symptom severity vs. patient benefit, and (pre-)medication with for example acetaminophen, nonsteroidal anti-inflammatory drugs, anti-histamines or corticosteroids should be evaluated (100). If allergic-type reactions, such as IgE-mediated or complement-mediated reactions are suspected, it is advisable to exclude patients with a history of allergies to biotherapeutics and/or formulation components, and plan for *ad hoc* sample collection in the protocol. In instances where hypersensitivity is observed, *ad hoc* sampling is necessary to investigate whether ADA formation is the cause, particularly for Type I and III reactions (2). In these cases, co-medication of anti-histamines to inhibit mast cell and basophil activation in IgE-mediated allergy risk or anti-cytokine (receptor) antibodies to avoid ADA-mediated cytokine release may also be considered.

When IG is observed during clinical development, impacting efficacy or safety, mitigation depends on the IG category. For life-threatening diseases without alternative treatment options, assessed as high IG risk, discontinuing treatment for patients with ADAs impacting efficacy or safety could result in disease progression which may be fatal. The mitigation strategy in such cases may involve high dose drug administration regimens for immune tolerance induction, or considering concomitant immunosuppressive treatment (5). As general immune suppressing co-medication, for instance, methotrexate or B-cell depleting agents (e.g. anti-CD20 therapeutics) may be used with or without additional immunomodulators (101). While these therapies have demonstrated effectiveness, they carry significant risk, including primary infections, reactivation of infections and cancer. Recently, novel approaches for targeted or antigen-specific tolerance strategies have gained attention. These include nanoparticle-mediated delivery of immunosuppressives, such as dexamethasone or rapamycin (ImmTOR) (101, 102). For life-threatening diseases with alternative treatment options, assessed as moderate risk, the mitigation strategy often involves treatment discontinuation for patients not showing therapy benefit to allow them to move to alternative medication. However, ADA information for decision making may not always be available.

Overall, it is essential to adopt a life cycle management approach to IRA that begins in the early stages of product development, extends through the initiation of clinical development, and continues through the Biologics License Application (BLA), remaining relevant even after market approval, in line with FDA guidance (6). While periodical updates throughout clinical development are not required, it is advisable to update the IRA at least prior to the start of pivotal studies, and to align the monitoring strategy with HAs, for example at the end-of-Phase-II-meeting. This ensures that the IG mitigation and monitoring strategy is suitable for addressing the relevant IG risk factors of a biotherapeutic.

## 10 Documentation of the immunogenicity risk assessment process

The IRA is a crucial component of the Integrated Summary of Immunogenicity (ISI), which is submitted to HAs during the marketing application. While the concept of applying a risk-based approach to evaluate the unwanted IG of biotherapeutics is widely recognized, conveying this information effectively in regulatory submissions poses challenges due to the necessity of consolidating numerous pieces of data. Regulatory agencies (FDA and EMA) recommend to provide brief summaries of the IG results in relevant places in electronic Common Technical Document (eCTD) section 2.7 (6) or 2.7.2.4 (5) and the full ISI report in section 5.3.5.3 (5, 6). Comprehensive recommendations on the structure of the ISI have been provided (5, 6, 103). Briefly, a proposed model format for the ISI consists of different sections tailored to the product and population summarizing the IRA, bioanalytical testing strategy, clinical study design and sampling strategy, results of clinical IG data analysis, followed by the conclusion of how IG affects the safety and efficacy of the biotherapeutic for the subject population.

According to the FDA guidance from 2019, it is also advised to include the IRA within the Investigational New Drug (IND) submission (6). However, limited guidance is available on the level of detail required for data presentation. The EIP acknowledges different approaches across pharmaceutical industry ranging from presenting a summary of the IRA placed in different sections of the IND document (e.g. 2.6.4., 2.5 or 2.7), to submitting a full IRA document in section 5.3.5.3.

## 11 Immunogenicity risk assessment in biosimilar development

The IRA for a biosimilar, in general, can follow the same principles as innovative therapeutic proteins (104). The common approach for novel biotherapeutics is performing an IRA at an early stage of development with a multidisciplinary team, due to the lack of any clinical experience, to understand which factors contribute to the likelihood and the impact of the ADA response. However, one major difference in biosimilar development is that published clinical IG data are available for the reference product (RP) that can directly inform on the patient and disease-related IG risks. If a comprehensive and robust comparative analytical assessment demonstrates “high similarity” between the proposed biosimilar product and the RP with little or no residual uncertainty related to clinical IG, then this can also directly inform on product and process related IG risks. Since IG is part of demonstrating similarity, ADA collection and testing is generally required in clinical studies, irrespective of risk. Further, published IG data provides ADA and NAb incidences and, in most cases, also the associated impact on safety and efficacy. Publicly available PK data for the RP can be used, in addition, to appropriately advise on a clinical IG monitoring strategy for the biosimilar development program. Nevertheless, the quality and quantity of information regarding the IG can vary greatly depending on the RP, i.e. details on ADA magnitude and whether they are transient or persistent are often not reported. In some cases, if only the number of positive/negative subjects is reported, data on the clinical impact of the IG may be limited. Thus, the information on the clinical relevance of the IG is often inconsistent for marketed biologics (105). Although the implementation of an IRA in biosimilar development is optional, it can be supportive to assess specific parameters which may slightly differ from biosimilar candidate to the RP and to determine the extent of required IG data (e.g. by sampling frequency and duration of clinical study/required follow-up period) to compare IG of the biosimilar and RP in relation to clinical end points. Hence, the comprehensiveness of an IRA may vary among different molecules. In addition, an IRA may be warranted in situations where the expression system and formulation is different from the RP. There is also a regulatory expectation that the biosimilar companies use state-of-the-art bioanalytical assays to generate meaningful comparative IG data, which could deviate from the original data reported by the RP, i.e. when using different bioanalytical assays. So, depending on the ADA characterization, appropriateness of the ADA assays and ADA impact on PK, efficacy and safety reported for the RP, a formal IRA of the proposed biosimilar (also may include clinical IG of the RP) may be considered and included as part of the biosimilar IND or clinical trial application dossier.

Since all clinical studies include the RP as a direct comparator to the biosimilar and assess the IG utilizing the current state-of-the-art bioanalytical assays using a single assay, it is not unexpected to observe differences in IG data to the RP reported in the drug label. But the biosimilar concept focuses on demonstrating similarity to its reference(s). In most cases a validated ADA method with a three-tiered testing approach (screening, confirmatory, titer (or S/N)) and a NAb assay are used in all clinical studies independent of the assigned risk category. Analytical assays capable of detecting (binding and neutralizing) antibodies against both the biosimilar and the RP in the same manner (one assay approach) are preferred, provided antigenic equivalence is demonstrated during ADA and NAb assay validation (106). Importantly, the ADA sampling schedule should allow the assessment of transient and persistent ADA responses, if appropriate, the magnitude of the ADA positive samples and most important, the overall comparative evaluation of any potential impact of IG on PK, PD (if applicable), efficacy and safety. As per default, all ADA samples collected within a study should be analyzed, unless there is a prior alignment with HAs. An essential element of this comparability exercise and the designation of a product as a biosimilar is to demonstrate that there are no clinically meaningful differences in the IG profile compared to the RP. As per regulatory guidelines, biosimilars undergo extensive comparability studies including physical, chemical, and biological characterization, as well as clinical trials. The evaluation of IG is usually performed in a PK comparability study, generally in healthy subjects, and, to the current state, in one or more comparative (parallel-arm) clinical studies, in a sensitive population to detect any IG differences if they exist between the market-licensed RP and the biosimilar candidate. Upon completion of the comparative clinical studies between the proposed biosimilar and the RP, there is a regulatory expectation that details on the IG incidence and the impact to exposure (PK), safety, and efficacy are provided. However, limited guidance is available on the IG data presentation and inclusion of the IRA. The EIP acknowledges there are different approaches across biosimilar companies. IG details can be included in the clinical study report(s) or alternatively a similar documentation approach as described for innovative biotherapeutics in the previous section can be used.

## 12 Conclusion

After years of extensive experience, the EIP fosters a common understanding of the IRA process for biotherapeutics within pharmaceutical industry and reinforces its importance during development. The current publication enables teams to conduct the IRA by integrating regulatory recommendations, recent literature examples, industry experience and business considerations into their evaluations. This allows to define tailored mitigation and monitoring strategies for low, moderate and high-risk programs, ensuring that safe and efficacious drugs reach patients. The IRA poses a significant challenge during the early development phases, particularly before the initiation of clinical trials. At this stage, considerable uncertainty exists in assigning the IG risk category to a biotherapeutic product, since the IG profile in the targeted patient population is unknown. Consequently, the EIP addresses that uncertainty by providing harmonized recommendations on risk classification based on the anticipated safety consequences evaluated in relation to disease severity and available treatment options. This evaluation is further supported by existing knowledge and, when no safety concerns are expected, by strategic business considerations. Additionally, the EIP highlights the impact of the IRA on early de-risking activities during molecular design and optimization, bioanalytical monitoring throughout development, and mitigation strategies during clinical trials. It emphasizes that the pharmaceutical industry employs early de-risking based on company strategy; for low-risk molecules, minimal action may be taken, while extended testing is pursued for higher-risk products. Furthermore, it notes that depending on anticipated safety consequences, analysis beyond traditional bioanalytical methods may be required for high-risk molecules in clinical trials, and that a high-risk designation may not always necessitate a NAb assay. The EIP also underscores the importance of maintaining an ongoing dialogue with HAs throughout development to ensure that an appropriate risk assessment strategy and methodologies are applied. By advocating for a unified approach to IG risk categorization, EIP paves the way for more streamlined IG testing strategies in future.

## Data Availability

The original contributions presented in the study are included in the article/supplementary material. Further inquiries can be directed to the corresponding author.
